# The spliceosomal autoantigen heterogeneous nuclear ribonucleoprotein A2 (hnRNP-A2) is a major T cell autoantigen in patients with systemic lupus erythematosus

**DOI:** 10.1186/ar2007

**Published:** 2006-07-19

**Authors:** Ruth Fritsch-Stork, Daniela Müllegger, Karl Skriner, Beatrice Jahn-Schmid, Josef S Smolen, Günter Steiner

**Affiliations:** 1 Division of Rheumatology, Department of Internal Medicine III, Medical University of Vienna, Austria; 2 Center of Molecular Medicine (CeMM) of the Austrian Academy of Sciences, Vienna, Austria; 3 Charité University Medicine Berlin, Department of Rheumatology and Clinical Immunology, Humboldt University and Free University, Berlin, Germany; 4 Institute of Pathophysiology, Medical University of Vienna, Austria; 5 Ludwig Boltzmann Institute for Rheumatology and Balneology, Vienna, Austria

## Abstract

A hallmark of systemic lupus erythematosus (SLE) is the appearance of autoantibodies to nuclear antigens, including autoantibodies directed to the heterogeneous nuclear ribonucleoprotein A2 (hnRNP-A2), which occur in 20% to 30% of SLE patients as well as in animal models of this disease. To investigate the underlying cellular reactivity and to gain further insight into the nature and potential pathogenic role of this autoimmune response we characterized the T cell reactivity against hnRNP-A2 in patients with SLE in comparison to healthy controls. Cellular proliferation of peripheral blood T cells to hnRNP-A2 was determined by [3H]thymidine incorporation and T cell clones (TCCs) specific for hnRNP-A2 were grown by limiting dilution cloning; IFNγ, IL-4 and IL-10 in culture supernatants were measured by ELISA. Bioactivity of culture supernatants was determined by incubation of anti-CD3/anti-CD28 stimulated peripheral blood CD4+ T cells with supernatants of TCCs. Stimulation assays performed with peripheral blood mononuclear cells of 35 SLE patients and 21 healthy controls revealed pronounced proliferative responses in 66% of SLE patients and in 24% of the controls, which were significantly higher in SLE patients (p < 0.00002). Furthermore, hnRNP-A2 specific TCCs generated from SLE patients (*n *= 22) contained a relatively high proportion of CD8+ clones and mostly lacked CD28 expression, in contrast to TCCs derived from healthy controls (*n *= 12). All CD4+ TCCs of patients and all control TCCs secreted IFNγ and no IL-4. In contrast, CD8+ TCCs of patients secreted very little IFNγ, while production of IL-10 did not significantly differ from other T cell subsets. Interestingly, all CD8+ clones producing IL-10 in large excess over IFNγ lacked expression of CD28. Functional assays showed a stimulatory effect of the supernatants derived from these CD8+CD28- hnRNP-A2 specific TCCs that was similar to that of CD4+CD28+ clones. Taken together, the pronounced peripheral T cell reactivity to hnRNP-A2 observed in the majority of SLE patients and the distinct phenotype of patient-derived CD8+ TCCs suggest a role for these T cells in the pathogenesis of SLE.

## Introduction

Systemic lupus erythematosus (SLE) is an autoimmune disease characterized by a wide spectrum of multi-organ manifestations, and genetic, hormonal, environmental and immunoregulatory factors are known to contribute to expression of the disease [1]. However, in spite of the considerable accumulated knowledge, the detailed etiopathogenesis of SLE still remains elusive. The presence of autoantibodies (autoAbs) to nuclear antigens in virtually all SLE patients constitutes the most characteristic serological feature of this disorder. Among the numerous autoantigens described, the nucleosome represents an important target structure of the autoimmune attack, leading to the formation of a wide array of autoAbs directed to single histones, histone-DNA complexes and double-stranded (ds)DNA (reviewed in [2,3]). The search for new serological markers has led to the identification of a number of additional autoantigens, among them the Ro and La proteins [4], as well as components of the spliceosome. This large and highly dynamic complex contains many evolutionarily conserved proteins, such as the U1–70 kD RNP or the Sm proteins [5,6], which are targeted by approximately 30% of SLE patients.

About 15 years ago, the heterogeneous nuclear ribonucleoprotein (hnRNP-)A2, another component of the spliceosome, was characterized as a novel target of autoAbs in systemic autoimmune diseases [7]. Although initially described to occur mainly in patients with rheumatoid arthritis (RA), autoAbs to hnRNP-A2 (originally termed anti-RA33) were later detected also in 20% to 30% of patients with SLE as well as in 40% of patients with mixed connective tissue disease, usually in association with other anti-spliceosomal autoAbs [8,9]. The autoAb response against hnRNP-A2 shows some differences in epitope recognition among patients with RA, SLE and mixed connective tissue disease [10]. Interestingly, autoAbs to hnRNP-A2 have also been detected in several animal models of RA and SLE [11,12]. HnRNP-A2 has a predominant nuclear localization and exerts multiple functions, including regulation of alternative splicing, transport of mRNA and regulation of translation [13-17].

Most of the autoAbs in SLE patients have undergone immunoglobulin class switching and affinity maturation. This and the association of HLA-DR subtypes with the presence of certain autoAbs indicates an antigen-driven immune response, emphasizing the role of T cells in SLE [18,19]. The cellular aspect of the immune response to DNA in its various forms has been extensively studied, including the characterization of T cells inducing anti-dsDNA autoAb production by B cells [20,21]. Interestingly, most of the T cell clones (TCCs) raised from SLE patients were of the Th1 or Th0 subset [21,22]. However, there have been only few reports about the T cell response to spliceosomal antigens, mainly focusing on the T cell reactivity to small nuclear ribonucleoprotein antigens [23]. In a recent report by Greidinger and colleagues, the authors characterized hnRNP-A2 specific CD4+ TCCs derived from one mixed connective tissue disease patient and two SLE patients, and attributed a possible pathogenic role to these T cells in SLE [24]. However, the presence of autoreactive T cells is not limited to patients, but has repeatedly been observed also in healthy individuals [21,25,26]; thus, the search for possible differences in autoantigen specific cellular reactivity between patients and healthy controls might give insight into the pathogenesis of the respective autoimmune disease.

Recently, we were able to characterize the cellular response against hnRNP-A2 in patients with RA [27]. We observed that approximately half of the RA patients harbor T cells against hnRNP-A2. In accordance with the perception of RA as an inflammatory, Th1 type systemic autoimmune disease, all generated TCCs were of the Th1 subtype, as defined by their predominant IFNγ secretion.

To gain more insight into the role of autoantigen specific T cells in SLE, we investigated spontaneous T cell responses to hnRNP-A2 in patients with SLE and in healthy control subjects and characterized TCCs specific for this antigen. The data obtained suggest that hnRNP-A2 may constitute an important T cell autoantigen in patients with SLE, indicating a potential role for it in the pathogenesis of this disorder.

## Materials and methods

### Patients and controls

Peripheral blood from 35 patients with SLE (31 female, 4 male, mean age 36.7 ± 3.4 years, for demographic characteristics see Table 1) classified according to the revised criteria of the American College of Rheumatology [1] was drawn into heparinized test tubes. Informed consent was obtained from all patients. Most patients were treated with immunomodulatory drugs (*n *= 21) and/or low-dose glucocorticoids (*n *= 15). Nine patients did not receive any medication. Disease activity was determined by European Consensus Lupus Activity Measurement (ECLAM) score [28]. While 87% of the patients had moderately active disease (ECLAM score <3), 5 patients had active SLE with an ECLAM score ≥3. The control population consisted of 21 healthy individuals (10 female and 11 male, mean age 31.8 ± 1.7 years).

### Antigens

Recombinant full-length hnRNP-A2 was used in all experiments. The cDNA encoding the antigen [15] was cloned into the pET-30 LIC vector (Novagen, Madison, WI, USA) and expressed as His-tagged fusion protein as described [27]. Purification from bacterial lysates was achieved by Ni-NTA affinity chromatography (Quiagen, Hilden, Germany) followed by Polymyxin B Sepharose adsorption (BioRad, Hercules, CA, USA) and anion exchange chromatography on DEAE Sepharose (Pharmacia, Uppsala, Sweden) essentially as described [27]. Endotoxin content was determined by the lympholytic amoebocyte lysate assay (BioWhittaker, Verviers, Belgium). Using this procedure, a more than 99% pure, endotoxin-free preparation was obtained. The optimum concentration for proliferation assays was found to be 0.35 μg/ml. Tetanus toxoid as control antigen was obtained from Pasteur Merieux Connaught (Willowdale, Ontario, Canada) and used at a concentration of 0.5 U/ml as previously described [29].

### Dectection of antibodies and cytokines

AutoAbs to hnRNP-A2 were detected by immunoblotting as described [8,10], employing the recombinant antigen and additionally by ELISA (IMTEC, Berlin, Germany). Cytokines were measured by ELISA (BioSource, Fleurus, Belgium) in supernatants of TCCs after 24 hours incubation with 0.35 μg/ml hnRNP-A2 or 0.5 U/ml tetanus toxoid as control antigen. Detection limits were 9 pg/ml for IFNγ, 4 pg/ml for IL-4, and 8 pg/ml for IL-10.

### T cell stimulation assays

Peripheral blood mononuclear cells (PBMCs) were isolated from heparinized blood of SLE patients and controls by centrifugation on Ficoll Hypaque (Pharmacia). After washing and counting cells were either immediately used or frozen in RPMI medium containing 10% dimethylsulfoxide and 20% fetal calf serum. Cells were cultured in the presence of the antigens for 5 days at 37°C in triplicate in 96-well plates (Costar, Cambridge, MA, USA) in a total volume of 200 μl (10^5^cells/well). Culture medium consisted of Ultra Culture serum-free medium (Biowhittaker, Wakersville, MD, USA) containing 2 mM glutamine and 0.02 mM 2-mercaptoethanol supplemented with 100 U/ml penicillin/streptomycin (Life Technologies, Paisley, UK). Phytohemagglutinin (Life Technologies) and IL-2 (Roche Molecular Biochemicals, Mannheim, Germany) were used as polyclonal stimuli. During the last 16 hours of culture 0.5 μCi per well [^3^H]TdR (Amersham-Pharmacia Biotech Europe, Freiburg, Germany) was added and the incorporated radioactivity was measured by scinitillation counting. Results were expressed as stimulation index (SI) defined as the ratio of mean counts per minute (cpm) obtained in cultures with antigen to mean cpm obtained in cultures incubated in the absence of antigen. An SI ≥3.0 and a Δcpm >1,000 (mean cpm obtained in cultures with antigen minus mean cpm obtained in cultures incubated in the absence of antigen) was regarded as a positive response.

### Antigen-specific T cell lines and clones

Antigen specific T cell lines were obtained using a previously established protocol [30]. In brief, 2 × 10^6 ^PBMCs were stimulated with 0.35 μg/ml hnRNP-A2 for 5 days in 24-well flat-bottomed culture plates. On the 5th day of culture, IL-2 was added at 20 U/ml and the culture was continued for an additional 7 days. To generate TCCs, T cell lines were restimulated with hnRNP-A2 and after 2 more days viable T cells were separated by Ficoll/Hypaque and seeded in limiting dilution (0.5 cells/well) in 96-wells plates. T cells were cultured in the presence of 1 × 10^5 ^irradiated (5,000 rad) allogeneic PBMCs as 'feeder cells', 0.5 μg/ml phytohemagglutinin and 20 U/ml IL-2 in medium containing 1% heat-inactivated human AB serum. Growing microcultures were then expanded at weekly intervals with fresh feeder cells in the presence of IL-2. The specificity of TCCs was assessed by proliferation assays incubating 2 × 10^4 ^T cells with 0.35 μg/ml hnRNP-A2 in the presence of 10^5 ^autologous irradiated PBMCs. After 48 hours incubation and pulsing with [^3^H]TdR for an additional 16 hours, cells were harvested and the incorporated radioactivity was measured by scintillation counting. Production of IL-4, IFNγ and IL-10 was measured by ELISA in supernatants collected after 24 hours of incubation as described [27].

For phenotyping, cloned T cells (0.5 to 1 × 10^5^) were washed twice with ice-cold FACS buffer (phosphate-buffered saline, 5% fetal calf serum, 0.01% NaN_3_) and incubated for 30 minutes at 4°C with a fluorescein isothiocyanate (FITC) or phycoerythrin-conjugated monoclonal Ab (mAb) (BD Pharmingen, San Diego, USA). Anti-T cell receptorα/β, anti-CD4 and anti-CD28 mAbs were FITC-conjugated, and anti-T cell receptor γ/δ and anti-CD8 mAbs were phycoerythrin-conjugated. Antibodies of the appropriate IgG isotypes were used as negative controls. Afterwards cells were washed again in FACS buffer and analyzed with a FACScan flow cytometer (Becton Dickinson, Franklin Lakes, NJ, USA). The acquired data were analyzed using Flow-Jo software (Tree Star, Inc., Ashland, OR, USA).

### Stimulation assays with supernatants from T cell clones

To investigate a possible functional difference between the CD4+CD28+ and CD8+CD28- TCCs, purified CD4+ T cells from two SLE patients and two healthy controls (10^5 ^cells/well) were stimulated with platebound anti-CD3 mAb (clone OKT3, Janssen-Cilag, Saundertown, UK) and anti-CD28 mAb (clone 15E8, Caltag, Burlingame, CA, USA) and incubated in duplicates with either 50 μl supernatant derived from CD4+CD28+ or CD8+CD28- TCCs, respectively. After 24 hours, cells were pulsed with 0.5 μCi per well [^3^H]TdR and proliferation was measured after anadditional 16 hours. Incubation with IL-10 alone (0.5 to 20 ng/ml; Insight Biotechnology Ltd, Wembley, UK) was used as control.

### Statistical analysis

Unless stated otherwise, SI are expressed as mean ± SEM of the respective cohort analyzed. Student's t-test was used to determine differences between controls and SLE patients. A p value <0.05 was regarded significant. For assessment of correlations the Pearson's correlation coefficient was used.

## Results

### Proliferative responses to hnRNP-A2 in SLE patients and healthy controls

**Figure 1:**
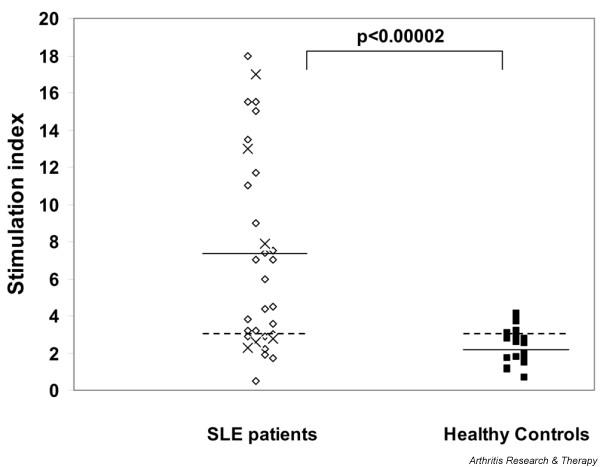
Proliferative responses of peripheral blood mononuclear cells (PBMCs) to heterogeneous nuclear ribonucleoprotein (hnRNP)-A2 in systemic lupus erythematosus (SLE) patients and healthy controls. Proliferation was measured by [^3^H]thymidine incorporation in PBMCs of 35 SLE patients and 21 controls in the presence or absence of hnRNP-A2. A stimulation index ≥3.0 and a Δcpm >1,000 cpm was considered a positive response (represented by the dashed line). The mean stimulation index (SI) was 6.7 ± 2.3 for SLE patients, and 2.3 ± 0.2 for controls (indicated by the solid bars). The difference between SLE patients and controls was highly significant (p < 0.00002). HnRNP-A2 seropositive SLE patients are indicated by crosses, and hnRNP-A2 seronegative SLE patients by diamonds.

Cellular reactivity against hnRNP-A2 was investigated by measuring proliferation of PBMCs obtained from 35 SLE patients and 21 age and sex matched healthy controls. Significant responses (defined as SI ≥3, Δcpm ≥1,000) were observed in 66% of the SLE patients and 24% of the controls (Figure 1). In SLE patients, the SI ranged from 0.5 to 18 (median SI = 4.4) with a mean SI of 6.7 ± 2.3, wheras the SI was much lower in the control group, ranging from 0.7 to 4.1 (median SI = 2.0) with a mean SI of 2.3 ± 0.2 (p < 0.00002). Moreover, a SI >4 was seen in 18 SLE patients (52%) but in only one control subject (5%).

As seen in Figure 1, T cell responses of SLE patients did not correlate with the presence of anti-hnRNP-A2 autoAbs, which were detected in 20% of the SLE patients but not in healthy controls, in accordance with previous observations [8,10]. There was also no correlation with anti-dsDNA antibodies or disease activity as measured with the ECLAM score.

The response to tetanus toxoid as control antigen was measured in 21 of the SLE patients and 16 of the healthy controls. Although stimulation elicited by this recall antigen was somewhat higher in SLE patients than in controls (6.2 ± 1.1 versus 5.0 ± 1.4), the difference was not significant. Of note, while in controls the stimulation elicited by tetanus toxoid was considerably higher than the one induced by hnRNP-A2, the response of SLE patients to tetanus toxoid was slightly but significantly lower than the response to hnRNP-A2 (Table 2).

### Generation of hnRNP-A2 reactive T cell clones

**Figure 2:**
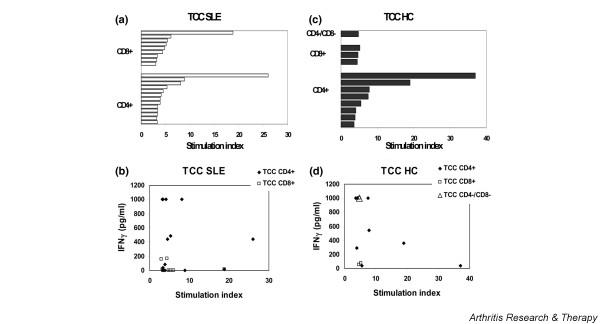
Phenotype, proliferation and cytokine production of heterogeneous nuclear ribonucleoprotein (hnRNP)-A2 specific T cell clones (TCCs) of systemic lupus erythematosus (SLE) patients and controls. TCCs were generated by limiting dilution and their specificity was assessed by measuring proliferation in response to hnRNP-A2. Cytokine production of TCCs was measured in the supernatants after 24 hours of stimulation with autologous antigen presenting cells and hnRNP-A2. **(a) **Proliferation of CD4+ and CD8+ TCCs of SLE patients. **(b) **Proliferation and IFNγ production of TCCs of SLE patients. **(c) **Proliferation of CD4+ and CD8+ TCCs of healthy controls (HCs). **(d) **Proliferation and IFNγ production of TCCs of healthy controls. There was no correlation between proliferative capacity and cytokine secretion.

T cell lines specific for hnRNP-A2 were established from the peripheral blood of 8 SLE patients and 4 healthy controls. Using the limiting dilution technique, between 1 and 6 antigen specific TCCs per individual (34 TCCs in total) could be raised. Twenty-two TCCs were derived from SLE patients: 13 (59%) of them were CD4+/CD8- and thus belonged to the helper T cell subset, while 9 were CD4-/CD8+. The TCCs showed high variability in their degree of proliferation, with SIs ranging from 3.0 to 26.0 and a mean SI of 6.2 ± 1.2. (Figure 2a). Analysis of the cytokine secretion pattern revealed pronounced IFNγ production by 10 of the clones, while the remaining 12 clones produced very little, if any, IFNγ. Cytokine production did not correlate with the degree of proliferation as even poorly proliferating TCCs secreted high amounts of IFNγ and vice versa (Figure 2b), whereas IL-4 was not detectable at all.

**Figure 3:**
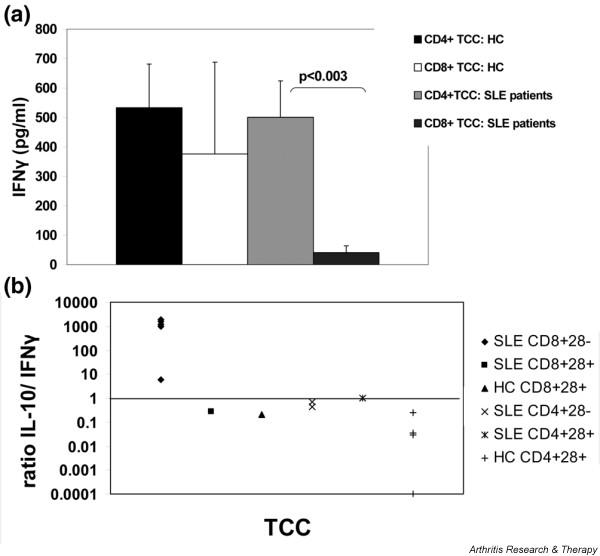
Cytokine production by heterogeneous nuclear ribonucleoprotein (hnRNP)-A2 specific T cell clones (TCCs) from systemic lupus erythematosus (SLE) patients and healthy controls **(a) **IFNγ production. Whereas IFNγ production of CD4+ and CD8+ TCCs derived from healthy controls was similar (mean ± SEM of 533 ± 148 for CD4+ versus 376 ± 312 for CD8+; p = not significant), IFNγ production by CD4 + TCCs from SLE patients was significantly higher than IFNγ production by patient derived CD8+ TCCs (500 ± 124 versus 40 ± 23 pg/ml; p < 0.003). **(b) **Ratio of IL-10/IFNγ secretion in hnRNP-A2 specific TCCs from SLE patients and controls. IL-10 secretion was measured in supernatants of 11 TCCs from SLE patients and 5 TCCs from healthy controls. IL-10 secretion largely exceeded IFNγ production in CD8+CD28- TCCs, which were all derived from SLE patients.

Proliferation of the 12 TCCs derived from control individuals was comparable to patient-derived clones, with SIs ranging from 3.6 to 37 and a mean SI of 9.0 ± 2.8 (Figure 2c). Phenotyping, on the other hand, revealed some differences. The majority of TCCs (*n *= 8; 67%) were CD4+/CD8-, while 3 clones (25%) were CD4-/CD8+ and one TCC was CD4-/CD8-. In contrast to SLE TCCs, all clones secreted IFNγ, though in highly varying amounts (Figure 2d). Whereas in SLE patients IFNγ secretion was significantly higher in CD4+ than in CD8+ TCCs (p < 0.003), no difference in cytokine production was found in CD4+ and CD8+ TCCs from healthy controls (Figure 3a). Additionally, IFNγ secretion of CD8+ SLE TCCs was markedly lower than in CD8+ control TCCs (Figure 3a); however, due to the small number of CD8+ control clones (*n *= 3), the difference between patient and control TCCs (39.5 ± 29.4 pg/ml versus 376 ± 312 pg/ml) did not reach statistical significance.

**Figure 4:**
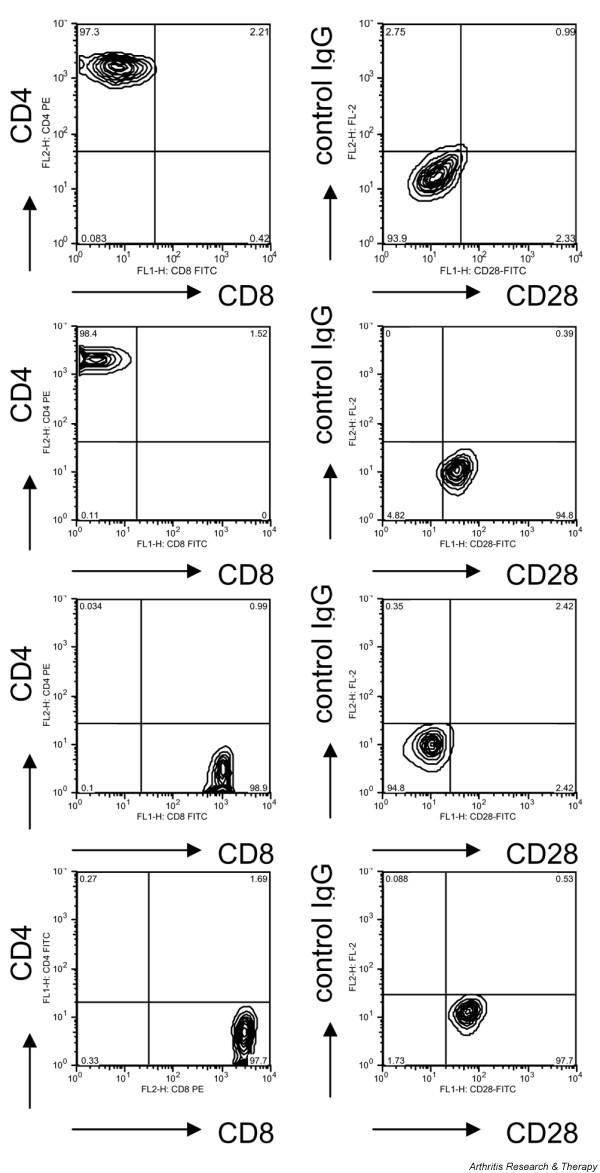
FACS analysis of four heterogeneous nuclear ribonucleoprotein (hnRNP)-A2 specific T cell clones (TCCs) derived from systemic lupus erythematosus (SLE) patients. Left panels: clones were stained with fluorescein isothiocyanate (FITC)- or phycoerythrin-conjugated mAbs against CD4 or CD8, respectively. Right panels: clones were stained with a FITC-conjugated anti-CD28 mAb and a phycoerythrin-conjugated control mAb. Top panel: FACS profile of a CD4+ CD28- TCC. Second panel: FACS profile of a CD4+CD28+ TCC. Third panel: FACS profile of a CD8+ CD28- TCC. Bottom panel: FACS profile of a CD8+ CD28+ TCC.

Expression of the costimulatory molecule CD28 was measured in six CD4+ and seven CD8+ TCCs from SLE patients and in five TCCs from healthy subjects (Figure 4): while all control TCCs examined (four CD4+ and one CD8+) expressed CD28, only three SLE TCCs were CD28+ (two CD4+ and one CD8+ TCC). Of note, all CD28+ clones and also the four CD4+CD28- SLE TCCs produced IFNγ, whereas this cytokine was barely detectable in the supernatants of the six CD8+CD28- TCCs; on the other hand, all clones secreted comparable amounts of IL-10. Thus, IL-10 was produced in 6- to 1,000-fold excess over IFNγ by the CD8+CD28- TCCs while all other clones examined (i.e. CD8+CD28+, CD4+CD28+ and CD4+CD28-) secreted comparable amounts of IFNγ and IL-10 (Figure 3b).

### Stimulation assays with supernatants of T cell clones

**Figure 5:**
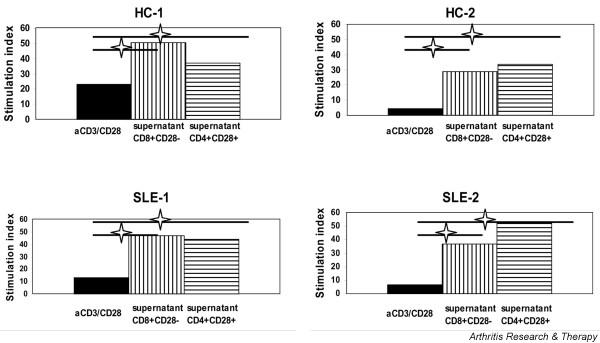
Proliferative responses of CD4+ T cells upon incubation with supernatants from T cell clones (TCCs). CD4+ T cells of two healthy controls (HC-1, HC-2) and two systemic lupus erythematosus (SLE) patients (SLE-1, SLE-2) were stimulated with platebound anti-CD3/anti-CD28 mAbs. Cells were incubated for 24 hours with the supernatants of a CD4+CD28+ and a CD8+CD28^- ^TCC derived from an SLE patient, and proliferation was measured after an additional 16 hours. The increase in proliferation was statistically significant for all supernatants examined (indicated by a star).

As pronounced differences in the cytokine secretion pattern of SLE patient derived CD4+CD28+ and CD8+CD28- TCCs had been observed, we were interested in the functional properties of these two populations. To address this question, CD4+ T cells (obtained from two SLE patients and two healthy controls) were stimulated with anti-CD3 and anti-CD28 mAbs and subsequently incubated with supernatants from two CD8+CD28- and two CD4+CD28+ TCCs derived from SLE patients (Figure 5). All supernatants caused a significant increase of the anti-CD3/anti-CD28 induced proliferative response: supernatants from CD8+CD28- clones enhanced proliferation by 452 ± 103% (p < 0.001), and CD4+CD28+ derived supernatants enhanced proliferation by 522 ± 161% (p < 0.02). Since the CD8+CD28- clones produced IL-10 in large excess over IFNγ, we examined the effect of IL-10 on proliferation: a modest reduction of proliferation was reproducibly seen, which, however, was not statistically significant (16.6 ± 6.6%, p = 0.07). Therefore, the pronounced stimulatory capacity of these supernatants cannot be attributed to IL-10 or IFNγ. Unfortunately, the TCCs were short-lived, limiting the number of experiments, and additional studies will be required to further investigate this issue.

## Discussion

In previous investigations we characterized the humoral response to hnRNP-A2 in SLE patients and some clinical implications thereof [8,10,31,32]. In this study we examined SLE patients and healthy controls for the presence of autoreactive T cells to hnRNP-A2 to better understand the role of this autoimmune response in the pathogenesis of SLE. The data obtained show the existence of a pronounced cellular response to hnRNP-A2 in the majority of SLE patients, which was far more vigorous than in healthy controls. In contrast, the response to the control antigen tetanus toxoid was similar in patients and controls, demonstrating the specificity of the immune response towards hnRNP-A2. Moreover, the response of SLE patients to hnRNP-A2 was of similar magnitude, or even slightly higher, than the response to tetanus toxoid, which is a recall antigen eliciting a pronounced response in the majority of individuals tested.

The occurrence of autoreactive T cells in healthy individuals is a common finding that has previously been reported for antigens associated with SLE [21] as well as other autoimmune diseases like pemphigus foliaceus [25] and multiple sclerosis [26]. Thus, the presence of hnRNP-A2 auoreactive T cells in healthy controls by itself was not surprising; a striking difference, however, was the significantly higher strength of the cellular immune response observed in SLE patients, which may be considered an indication for pathogenic involvement of these autoreactive cells and/or a lack of counter-regulation in SLE patients.

Interestingly, no correlation of cellular reactivity to hnRNP-A2 with the appearance of the respective autoAbs in SLE patients could be observed. Thus, hnRNP-A2 appears to be a predominant T cell antigen, while the generation of autoAbs might represent a bystander phenomenon occurring in a subgroup of patients. However, recent data indicate that in SLE patients the humoral response to hnRNP-A2 fluctuates considerably and increases during flares. Therefore, the prevalence of these autoAbs may be considerably higher than previously assumed (unpublished observation).

Although the cellular reactivity to hnRNP-A2 appeared to be primarily a Th1 response, we observed a relatively high percentage of CD8+ TCCs in SLE patients. Of particular interest, most of these clones lacked CD28 expression and produced neither IFNγ nor IL-4, but did produce IL-10. This may indicate a special pathological role of this T cell subset because this phenotype appeared to be restricted to SLE patient derived TCCs. In previous reports, CD4+ T cells in SLE were shown to be of predominantly Th1 or Th0 subtypes [21,24]. Furthermore, autoreactive CD4+ TCCs from SLE patients and healthy controls showed similar cytokine production [22]. Whereas the relatively high proportion of hnRNP-A2 specific CD8+ TCCs might mirror the common understanding of a generally increased CD8:CD4 ratio in SLE patients [33-35], there are only anecdotal reports about autoreactive CD8+ TCCs in SLE [6] and their role is not entirely clear and may be quite complex indeed. Thus, on the one hand, the rather low IFNγ secretion of patient derived CD8+ TCCs might be due to inherent abnormalities of CD8+ (suppressor) T cell function in SLE, as shown recently by Filaci and colleagues [36]. On the other hand, these TCCs secreted considerable amounts of IL-10 and, in contrast to the TCCs derived from healthy controls, were CD28 negative. Recently, a new T suppressor population was defined, which could be generated *in vitro *from CD8+CD28- T cells [37]. This subset exerted its regulatory and suppressive function in a cell contact independent manner via secretion of IL-10, which is in line with previous reports attributing a regulatory function to CD8+CD28- T cells [38-40]. An increase of these cells in patients with SLE might thus constitute an effort of counter-regulation within the disturbed immune system of SLE patients.

Surprisingly, though, the supernatants of the hnRNP-A2 specific CD8+CD28- TCCs derived from SLE patients did not inhibit proliferation of CD4+ T cells, but instead enhanced anti-CD3/anti-CD28 induced stimulation, similar to supernatants derived from CD4+CD28+ TCCs. As IL-10 alone had an antiproliferative effect, the increased content of IL-10 in the supernatants of CD8+CD28- TCCs was obviously counteracted by other yet unknown stimulatory components of the supernatant, which remain to be identified. Of note, the lack of CD28 expression is also a hallmark of senescent T cells, which are known to be increased in various autoimmune diseases and chronic inflammatory conditions and may show a rather aggressive phenotype [41]. Therefore, it is conceivable that the CD8+CD28- TCC may be derived from the senescent T cell pool of SLE patients; this also correlates better with our data.

Elevated IL-10 serum levels have been reported in SLE patients and correlated with clinical and serological disease activity, especially anti-DNA antibody titres [42]. Several cell types have been implicated in the production of IL-10 [43] and augmented IL-10 secretion has been linked to autoAb production in a model where PBMCs from patients with SLE were transferred into mice with severe combined immunodieficiency [44]. So, altogether, the CD8+CD28- T cell subpopulation might enhance the inflammatory process in SLE patients by stimulation of B cells via IL-10. Unfortunately, because of the short life-span of the clones, additional functional assays could not be performed and remain the subject of future investigations.

Finally, the question remains why hnRNP-A2 is such a preferred target of the T cell response in SLE, while autoAbs occur less frequently and may represent an epiphenomenon of ongoing T cell autoreactivity. On the other hand, autoAb titers increase during disease flares (unpublished observation) and, importantly, autoAbs to hnRNP-A2 are, together with anti-Sm antibodies, among the earliest detectable autoAbs in MRL/lpr mice, where they were found to precede anti-dsDNA autoAbs [11]. HnRNP-A2 is a multifunctional protein that, in the nucleus, partially colocalizes with spliceosomal complexes [13,14], a preferred autoimmune target in SLE. Thus, the data obtained in the course of this study further strengthen the view of the spliceosome being one of the predominant autoimmune target structures in SLE, playing a presumably pivotal role in the pathogenesis of this disease, even though the reasons for this are still far from being fully understood.

## Conclusion

Our data reveal a pronounced T cell response against the autoantigen hnRNP-A2 in the majority of SLE patients in contrast to a scarcer and lower response in healthy subjects. Although this autoreactivity seemed to be mainly conferred by CD4+ T cells showing a Th1 phenotype, a newly defined hnRNP-A2 specific CD8+CD28- T cell-subset was observed in SLE patients. This subpopulation showed a predominant IL-10 secretion and a lack of IFNγ or IL-4 production. Our data suggest involvement of both populations in the complex and still incompletely understood pathogenesis of SLE, and warrant further investigations into the cellular aspects of this autoimmune response.

## Abbreviations

autoAb = autoantibody; cpm = counts per minute; ds = double stranded; ECLAM = European Consensus Lupus Activity Measurement; ELISA = enzyme-linked immunosorbent assay; FITC = fluorescein isothiocyanate; hnRNP = heterogeneous nuclear ribonucleoprotein; IFN = interferon; IL = interleukin; mAb = monoclonal antibody; PBMC = peripheral blood mononuclear cell; RA = rheumatoid arthritis; SI = stimulation index; SEM = standard error of the mean; SLE = systemic lupus erythematosus; TCC = T cell clone.

## Competing interests

They authors declare that they have no competing interests.

## Authors' contributions

RF participated in the design of the study, worked with the T cell cultures and drafted the manuscript. DM performed the T cell cloning and T cell assays. KS prepared the recombinant antigen. BJS participated in the design of the study. JS and GS conceived of the study, participated in its design and coordination and helped to write the manuscript. All authors read and approved the final manuscript.

## Figures and Tables

**Table 1:** Demographic characteristics of systemic lupus erythematosus patients

Patient	Sex	Age (years)	ECLAM score	Medication
1	Female	25	1.5	HQ,P
2	Female	25	0.5	0
3	Female	52	2.5	0
4	Female	24	1.5	C,P
5	Female	29	0	HQ
6	Female	43	1	0
7	Female	29	0.5	HQ,P
8	Female	38	1.5	HQ,Aza,P
9	Female	34	0	MTX,P
10	Female	60	0	MTX,P
11	Female	52	1	P
12	Female	48	2	HQ,P
13	Female	43	0	0
14	Female	62	1	HQ,P
15	Male	33	0	HQ
16	Female	34	2	Aza,P
17	Female	37	3	0
18	Male	30	0	Ara,P
19	Female	45	4	0
20	Female	31	1	0
21	Female	24	6	Aza,P
22	Female	30	1	0
23	Female	29	1.5	MTX,HQ
24	Female	49	2	0
25	Female	28	2	HQ
26	Female	24	1.5	P
27	Male	45	1	HQ,P
28	Female	22	3.5	HQ,P
29	Female	26	0	HQ
30	Female	29	1	P
31	Female	24	2	0
32	Female	61	2	P
33	Female	43	5.5	MTX,P
34	Female	33	1	0
35	Male	44	1	C

0, none; Aza, azathioprin; C, cyclophosphamide; ECLAM, European Consensus Lupus Activity Measurement; HQ, hydroxychloroquine; MTX, metothrexate; P, prednisolone.

**Table 2:** Proliferative responses to hnRNP-A2 and the control antigen tetanus toxoid in SLE patients and healthy controls

	Proliferative response to		
	hnRNP-A2	Tetanus toxoid	P value

SLE patients (*n *= 21)	7.5 ± 1.2	6.2 ± 1.1	P < 0.05
Healthy controls (*n *= 16)	2.5 ± 0.6	5.0 ± 1.4	P < 0.002
P value	P < 0.000002	NS	

Data are given as mean stimulation index ± SEM. Student's paired t-test was used to calculate statistical significances. hnRNP, heterogeneous nuclear ribonucleoprotein; NS, not significant; SLE, systemic lupus erythematosus.
